# A Retrospective Study of the Impact of Intraoperative Intact Parathyroid Hormone Monitoring During Total Parathyroidectomy for Secondary Hyperparathyroidism: STARD Study

**DOI:** 10.1097/MD.0000000000001213

**Published:** 2015-07-24

**Authors:** Takahisa Hiramitsu, Yoshihiro Tominaga, Manabu Okada, Takayuki Yamamoto, Takaaki Kobayashi

**Affiliations:** From the Department of Transplant and Endocrine Surgery, Nagoya Daini Red Cross Hospital (TH, YT, MO, TY); and Department of Transplant Immunology, Nagoya University School of Medicine, Showa-ku, Nagoya, Aichi, Japan (TK).

## Abstract

The study aimed to evaluate the diagnostic accuracy of intraoperative intact parathyroid hormone (IO-iPTH) in patients with secondary hyperparathyroidism (HPT).

The cut-off for IO-iPTH monitoring remains unknown.

This was a single-center retrospective review of 226 consecutive patients (107 males and 119 females) who underwent parathyroidectomy data for secondary HPT between May 2010 and March 2014. The predetermined cut-off for IO-iPTH was a 70% IO-iPTH drop from baseline 10 minutes after total parathyroidectomy and thymectomy. We used <60 pg/mL iPTH value on postoperative day 1 (POD1) as an indicator of successful removal of parathyroid glands and reviewed the frequency of reoperation other than in autografted sites during the observation period. This study was based on the Standards for the Reporting of Diagnositic accuracy compliant.

The reoperation rate in patients with >60 pg/mL iPTH value (POD1) was significantly higher than that in patients with <60 pg/mL iPTH value (POD1), (13.0% versus 0.5% *P* = 0.003). Sensitivity, specificity, and accuracy of >70% IO-iPTH drop were 97.5%, 52.2%, and 92.9%, respectively, this criterion was demonstrated to be beneficial in 26 patients. In 5 patients, <70% IO-iPTH drop was observed and further exploration enabled sufficient removal of parathyroid glands. In 21 patients, although fewer than 4 parathyroid glands were removed after enough explorations, >70% IO-iPTH drop enabled termination of operations and iPTH value (POD1) was <60 pg/mL.

An iPTH value of <60 pg/mL (POD1) was a good predictor for successful parathyroidectomy. A 70% IO-iPTH drop from the baseline was appropriate to determine sufficient parathyroid gland removal during parathyroidectomy for patients with secondary HPT.

## INTRODUCTION

Intraoperative intact parathyroid hormone (IO-iPTH) monitoring is common in parathyroidectomy for primary hyperparathyroidism (HPT), particularly for minimally invasive procedures, and several criteria have been established for the same.^1–3^ Marcin et al evaluated the efficacy of various IO-iPTH monitoring criteria and concluded that the Miami criterion of >50% drop from the highest intraoperative PTH values at 10 minutes after excision has the best sensitivity, specificity, and accuracy.^4^ Although several criteria have been proposed for IO-iPTH monitoring in patients with secondary HPT, no consensus has been reached. Using the Quick-Intraoperative Bio-Intact PTH assay (Nichols Institute Diagnostics, San Clemente, CA), a number of criteria for parathyroidectomy in patients with secondary HPT has been reported. This assay was quick and reliable, taking only 15 to 20 minutes to obtain results. Moreover, the established criteria were excellent, enabling the efficient removal of all parathyroid glands during parathyroidectomy for secondary HPT.^5–10^ Unfortunately, this kit is no longer available, therefore, iPTH is now widely used to evaluate the parathyroid function. A limitation of iPTH is that it consists of many fragments with varying half-lives and proportions.^11–13^ Consequently, IO-iPTH monitoring has rarely been reported.

For secondary HPT, successful parathyroidectomy should be performed to avoid continued stimulation by the residual gland tissue that might cause recurrent secondary HPT in the context of impaired renal function.^14^ However, embryological and anatomical features make it difficult to perform successful parathyroidectomy. Furthermore, in 13% of patients, more than 4 parathyroid glands exist, and ectopic glands exist in the thymus, mediastinum, thyroid gland, and upper neck.^15,16^ Thus, if the sufficient removal of parathyroid glands can be intraoperatively confirmed, it may be possible to avoid further unnecessary explorations and to prevent operative failure due to missed supernumerary glands.

To date, only a few studies on a small number of patients have reported criteria to determine operative success using IO-iPTH monitoring.^17–19^ Therefore, we aimed to retrospectively evaluate our IO-iPTH criteria for parathyroidectomy in a large number of patients with secondary HPT.

## MATERIALS AND METHODS

### Ethical Review

This study was approved by the institutional review board in Nagoya Daini Red Cross Hospital.

### Participants

We enrolled 226 consecutive patients (107 males and 119 females) who underwent parathyroidectomy for refractory secondary HPT between May 2010 and March 2014. All data were collected retrospectively.

## TEST METHODS

### Preoperative Diagnosis

Parathyroid glands were preoperatively located by ultrasound, computed tomography, or TechnetiumTc-99m sestamibi scan.

### Surgical Indications

Parathyroidectomy were basically adapted according to clinical practice guideline for the management of secondary HPT in chronic dialysis patients.^20^ However, patients whose iPTH were well treated under 500 pg/mL with calcimimetric agents were also adapted parathyroidectomy, if they were managed to take calcimimetric agents in spite of their bowel symptoms.

## OPERATIVE METHOD

All patients underwent total parathyroidectomy and transcervical thymectomy with forearm autograft under general anesthesia. Procedures were performed at a single center by 4 experienced surgeons who understood this criterion well. To confirm the parathyroid tissue, frozen sections of all resected specimens were examined by a pathologist during the operation. Thereafter, approximately 90 mg of the parathyroid tissue was autografted. After operation, paraffin sections were examined by a pathologist to determine the final number of removed parathyroid glands.

### IO-iPTH Measurement

We measured iPTH by St AIA-PACK IPTH (Tosoh Corporation, Tokyo, Japan), which is a two-site immunoenzymometric assay, using an iPTH immunoreaction reagent. With this assay, 1–34 and 39–84 amino acid regions of iPTH were targeted. After separating blood samples centrifugation, serum iPTH was bound with a polyclonal antibody immobilized on magnetic beads and enzyme-labeled polyclonal antibodies. The unbound magnetic beads were washed out 10 minutes after incubation. Next, the fluorogenic substrate, 4-methylumbelliferyl phosphate, was added for the enzyme substrate reaction at 37 °C. The converted 4-methylumbelliferone was measured and found to be proportional to the iPTH concentration. The results were obtained within 20 minutes. The measurable iPTH concentration was between 1.0 and 2000 pg/mL. Cross reactivity with other PTH fragments was as follows:

PTH (7–84) = 107.4%, PTH (1–34) < 0.02%, PTH (13–34) < 0.02%, PTH (39–84) < 0.02%, and PTH (53–84) < 0.02%.

### IO-iPTH Monitoring Protocol

Values of iPTH were measured on admission, during the procedure and on postoperative day 1 (POD1). Before the skin incision, baseline control blood samples were obtained from a peripheral artery for preoperative iPTH assessment (Pre-IO-iPTH). Blood was again extracted 10 minutes after total parathyroidectomy and thymectomy for postoperative iPTH assessment (Post-IO-iPTH). A >70% IO-iPTH drop 10 minutes after total parathyroidectomy and thymectomy, predicted operative success. The procedure was terminated even if fewer than 4 glands were excised after routine exploration. On the other hand, if IO-iPTH dropped <70% from Pre-IO-iPTH level, further exploration was performed and IO-iPTH protocol was repeated for each additional gland. To assess the successful removal of parathyroid glands, parathyroidectomy results were evaluated against iPTH values of <60 pg/mL on POD1. Serum calcium levels were adjusted according to the protocols. An iPTH value (POD1) of <60 pg/mL is an upper normal range that has been used as a predictive value for persistent and recurrent secondary HPT.^21^

### Serum Calcium Adjustment Between Operation and Blood Sampling on POD1

Serum calcium levels were routinely evaluated soon after the operations and 6 hours after the operations. And when patients showed hypocalcemic symptoms, serum calcium levels were also evaluated. Serum calcium levels were adjusted according to the following protocol:

1. serum calcium level > 4.5 mEq/L: observation.
2. 4.0 mEq/L < serum calcium level < 4.5 mEq/L without hypocalcemic symptoms: observation.
3. 4.0 mEq/L < serum calcium level < 4.5 mEq/L with hypocalcemic symptoms: intravenous calcium infusion.
4. serum calcium level < 4.0 mEq/L: intravenous calcium infusion.

### Indication for Reoperation

The patients who became refractory to medications were evaluated with ultrasound, computed tomography, or TechnetiumTc-99m sestamibi scan. In cases with residual parathyroid glands identified, reoperations were performed.

#### Statistical Methods

Patients were distributed into true positive (TP), true negative (TN), false positive (FP), and false negative (FN) (Table 1). Sensitivity, specificity, positive predicitive value (PPV), negative predicitive value (NPV), and accuracy were evaluated.

**TABLE 1:**
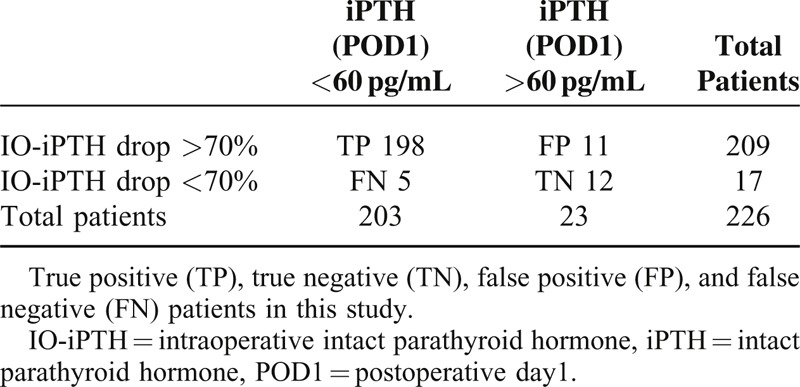
Accuracy of IO-iPTH Monitoring During Parathyroidectomy for Secondary Hyperparathyroidism

Values of iPTH on admission day were evaluated with Mann–Whiteney *U* test, whereas reoperation rates were evaluated with Chi-square test. For all the analyses, a *P* value of <0.05 was considered statistically significant. Analyses were performed using SPSS for Windows version 13.0 (IBM, Chicago, IL) statistical software.

## RESULTS

### Participants

The study population included a total of 226 patients (107 males and 119 females) with a mean age of 55.4 ± 12.4 years between May 2010 and March 2014. Median iPTH on admission day was 451 pg/mL. In 66 (29.2%) patients, the values were <300 pg/mL. The mean follow-up duration before recurrence was 33.4 ± 13.5 months.

### Test Results

iPTH values were measured during the operation and on POD1. Serum calcium levels were adjusted according to the protocols.

Comparing patients with an iPTH value (POD1) of <60 pg/mL and those with an iPTH value (POD1) of >60 pg/mL, iPTH on admission day did not differ significantly (895.6 ± 186.7 versus 592.6 ± 35.9 pg/mL, respectively, *P* = 0.072). No adverse event from performing IO-iPTH monitoring was identified.

### Estimates

#### Validity of an iPTH (POD1) value of <60 pg/mL

**FIGURE 1:**
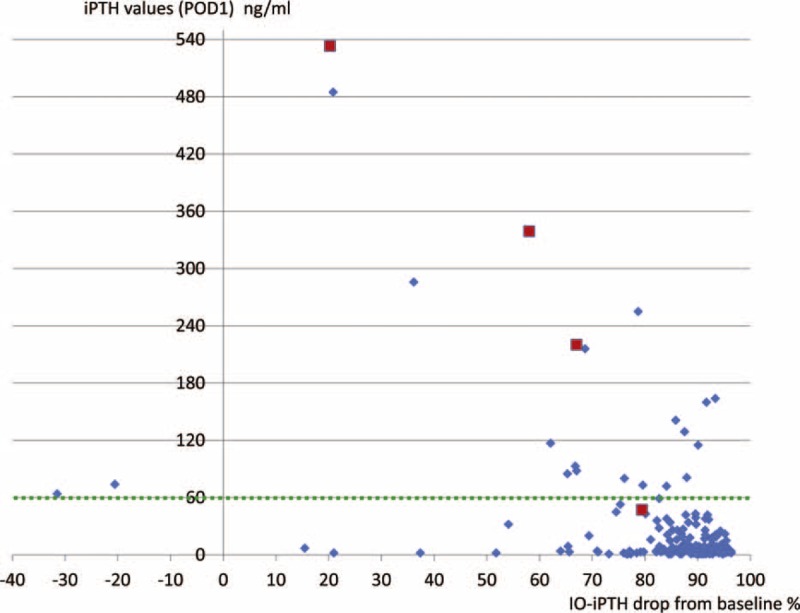
Trend of IO-iPTH levels in relation to drop in iPTH values from the baseline in patients who underwent reoperation. Patients who underwent reoperations. Patients who did not undergo reoperations. IO-iPTH = intraoperative intact parathyroid hormone, iPTH = intact parathyroid hormone, POD1 = postoperative day 1.

In 203 patients with iPTH values (POD1) of <60 pg/mL, only 1 (0.5%) patient underwent reoperation for the removal of a residual right lower parathyroid gland. Among 23 patients whose iPTH values (POD1) were >60 pg/mL, 3 (13.0%) underwent reoperation for the removal of a residual right upper gland, left lower gland, and mediastinal gland (Figure 1). There was a significant difference in the reoperation rates between the 2 groups divided according to the iPTH values (POD1, *P* = 0.003).

#### Diagnostic Accuracy of IO-iPTH Monitoring

In 209 patients (TP + FP), IO-iPTH dropped to >70%; and in 198 patients (TP), iPTH values (POD1) were <60 pg/mL. On the contrary, in 17 patients with negative results (FN + TN), IO-iPTH levels dropped to <70%, whereas in 5 patients (FN), iPTH values (POD1) were <60 pg/mL. iPTH values of <60 pg/mL on POD1 were identified in 203 patients (TP + FN), whereas in 23 patients (FP + TN), iPTH value (POD1) was >60 pg/mL. In 12 patients (TN), IO-iPTH dropped to <70% and iPTH values (POD1) were >60 pg/mL (Table 1).

Sensitivity, specificity, PPV, NPV, and accuracy of this criterion were 97.5% (95% CI 0.954–0.996), 52.2% (95% CI 0.418–0.726), 94.7% (95% CI 0.917–0.977), 70.6% (95% CI 0.489–0.923), and 92.9% (95% CI 0.896–0.962), respectively.

#### Patients Who Benefited From IO-iPTH Monitoring

**FIGURE 2:**
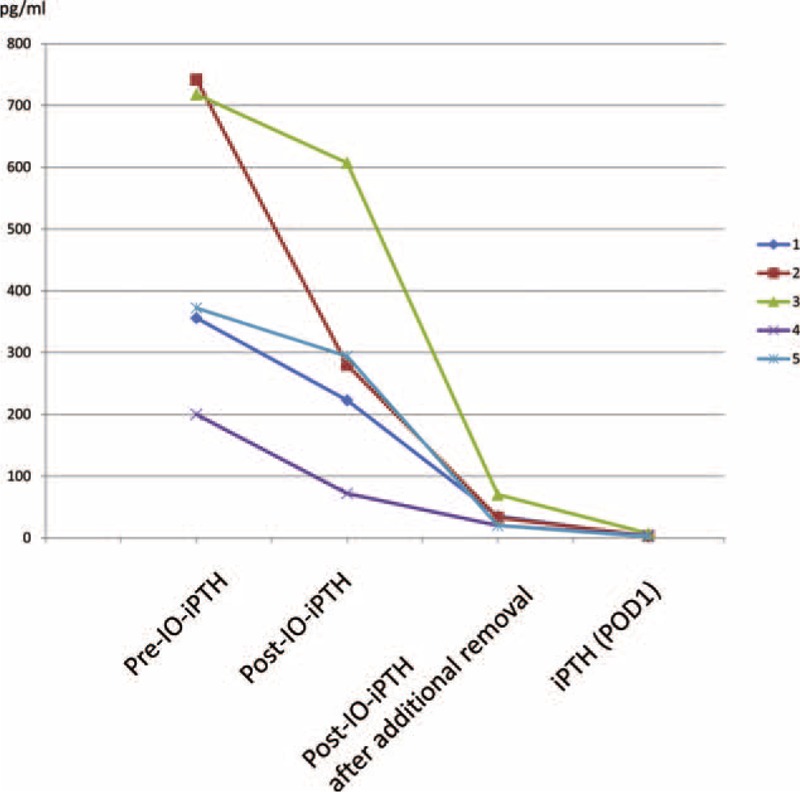
Benefit of IO-iPTH monitoring in patients. IO-iPTH = intraoperative intact parathyroid hormone, iPTH = intact parathyroid hormone, POD1 = postoperative day 1.

In 5 out of 198 patients with TP values, IO-iPTH dropped to <70% and further exploration enabled the removal of residual parathyroid glands (Figure 2). In 3 patients, additionally removed parathyroid glands were found in the usual location; however in 2 patients, locations were the intrathyroid and mediastinum (Table 2).

**TABLE 2:**
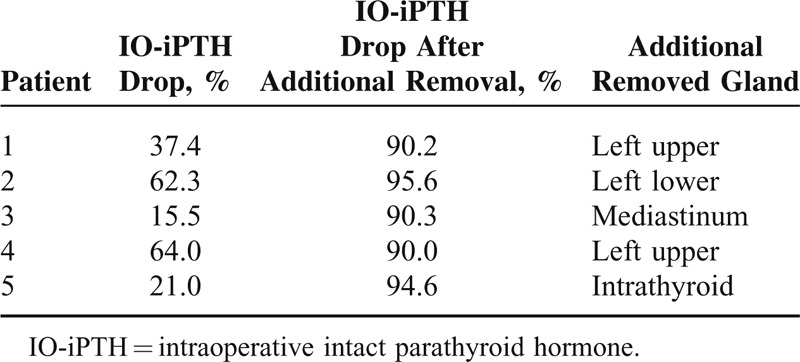
Benefits of IO-iPTH Monitoring During Parathyroidectomy for Secondary Hyperparathyroidism in 5 Patients

In 21 out of 198 patients with TP values, IO-iPTH dropped to >70% and this enabled us to avoid further explorations, although fewer than 4 parathyroid glands were identified during routine explorations (Table 3). In 7 out of 21 patients, fewer than 4 removed parathyroid glands were identified during the operation in the frozen section. However, in the paraffin section, more than 4 parathyroid glands were identified. In 5 out of 5 patients, parathyroid glands were microscopically identified in the thymus. In total, IO-iPTH monitoring was beneficial in 26 out of 198 (13.1%) patients.

**TABLE 3:**
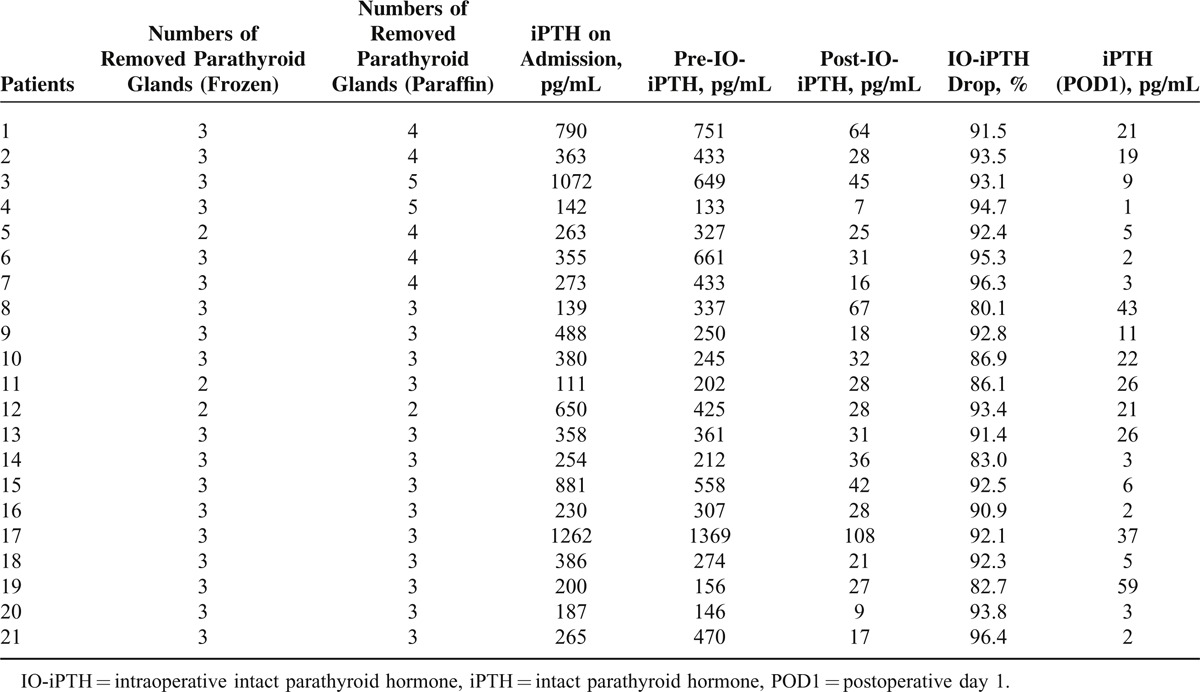
Benefits of IO-iPTH Monitoring During Parathyroidectomy for Secondary Hyperparathyroidism in 21 Patients

## DISCUSSION AND CONCLUSIONS

The criteria and efficacy of intraoperative PTH monitoring during parathyroidectomy for primary HPT had been well reported and established.^1–4^ On the contrary, there is no consensus yet on the criterion during parathyroidectomy for secondary HPT.

For the treatment of refractory secondary HPT, successful parathyroidectomy is essential in initial operation. Residual parathyroid glands may be stimulated when the renal function is impaired and may easily lead to recurrent secondary HPT. However, the number of parathyroid glands and existing lesions are different among individuals. These factors make it difficult to perform successful parathyroidectomy. Therefore, the confirmation of the sufficient removal of parathyroid glands during the operation is required. For these reasons, it is necessary to establish a criterion for IO-iPTH monitoring during parathyroidectomy for secondary HPT.

The criteria of Quick-Intraoperative Bio-Intact PTH assay (Nichols Institute Diagnostics) for parathyroidectomy of secondary HPT was well reported previously. The test was appropriate because it measured only whole PTH within 15 to 20 minutes. However, after the discontinuation of these assay kits, intact PTH assay kits have now been widely used.

iPTH consists of many kinds of fragments. C-terminal fragments are mainly secreted by glomerular filtration and accumulate in patients with impaired renal function.^11,12^ The half lives of C-terminal fragments are much longer in patients with impaired renal function.^22^ ST AIA-PACK intact PTH kit (Tosoh Corporation, Tokyo, Japan) is useful for iPTH measurement. However, whole PTH values may be overestimated.^22,23^ This complexity could make it more difficult to establish a criterion using iPTH than that using whole PTH. Thus, IO-iPTH monitoring with iPTH assay kit during parathyroidectomy for secondary HPT was rarely reported after the discontinuation of Quick-Intraoperative Bio-Intact PTH assay kit.

The efficacy of IO-iPTH monitoring during parathyroidectomy for secondary HPT was previously referred by Echenique Elizondo et al who investigated 25 patients with total parathyroidectomy.^17^ Their criterion was a 50% decrease in IO-iPTH values 10 minutes after removal, which was the same as the Miami criterion for primary HPT; in their study, all iPTH values at 24 hours became undetectable. However, this previous study included a small number of patients and no TN, FN, and FP cases were observed, therefore further investigations may be required. There were some other studies that investigated the appropriate criteria, but also with very limited number of patients.^18,19^ Thus, it was important to evaluate the efficacy of IO-iPTH monitoring in a larger study population.

This study was retrospective but included 226 consecutive patients. iPTH value (POD1) of <60 pg/mL is an upper normal value that has been used as a predictor of persistent and recurrent secondary HPT.^21^ In this study, an iPTH value (POD1) of >60 pg/mL was demonstrated as a good predictor for reoperation. Thus, it may be valuable to use this value as an indicator of successful parathyroidectomy.

In this study, the sensitivity, PPV, and accuracy of this criterion were excellent, that is, a >70% IO-iPTH drop can indicate successful parathyroidectomy, even in cases with fewer than 4 parathyroid glands identified during the operation. As reported, in 140 out of 902 (15.5%) patients with secondary HPT, supernumerary parathyroid glands existed in the thymus.^24^ In our usual procedure, transcervical thymectomy was performed in all patients, and small parathyroid glands in the thymus may be removed without identification during the operation. In our series, fewer than 4 parathyroid glands were removed and further unnecessary explorations were avoided in 21 patients. More than 4 parathyroid glands were identified in 7 out of 21 patients by paraffin sections. In 5 out of 7 patients, parathyroid glands were microscopically identified in the thymus. Therefore, thymectomy is an essential procedure for successful parathyroidectomy with IO-iPTH monitoring. On the contrary, it was proven that 3% to 6% of the general population had fewer than 4 parathyroid glands in the autopsy series.^15,25^ It may be reasonable in 14 patients, in whom fewer than 4 parathyroid glands were identified even in paraffin sections.

In patients with <70% IO-iPTH drop, further explorations and IO-iPTH monitoring 10 minutes after the removal of additional parathyroid glands were necessary. IO-iPTH monitoring should be repeated to confirm the sufficient removal of parathyroid glands. In our study, IO-iPTH dropped to <70% in 5 patients, and further exploration of the thymus and thyroid enabled us to find residual parathyroid glands. In these patients, IO-iPTH dropped to >70% after additional parathyroid glands were removed and indicated the successful removal of residual parathyroid glands. Nevertheless, if the IO-iPTH dropped to <70% and residual parathyroid glands were not identified in the usual location, such as the thymus, thyroid, or upper neck, even after the removal of more than 4 parathyroid glands, there was no further step but to evaluate the results with iPTH value (POD1).

On the contrary, specificity and NPV did not show excellent reliability in this criterion. It may be because of 16 FP and FN. FN in 5 patients may be accounted for by different half-lives and proportions of each iPTH fragments. As a result, IO-iPTH dropped to <70% at 10 minutes after removal, although iPTH value (POD1) was <60 pg/mL. In 11 FP patients, IO-iPTH dropped to >70%, but iPTH values (POD1) were >60 pg/mL. It seemed that sufficient parathyroidectomy had been performed during the operation, but residual parathyroid glands might still exist. Once IO-iPTH drops to >70%, it is impossible to reconfirm the removal of all parathyroid glands until POD1. This is one of the limitations of this criterion.

There are 3 pit falls of this criterion. First is the low specificity an NPV in despite excellent sensitivity, PPV, and accuracy. Second is that, in patients whose IO-iPTH dropped to <70% and whose residual parathyroid glands were not identified even after additional explorations, there were no further alternative procedures to search for residual glands. Lastly, it took at least 30 minutes to get results after total parathyroidectomy and thymectomy. If the additional removal of parathyroid glands is necessary, it will take at least 30 minutes more to confirm the sufficient removal of parathyroid glands. It may be time consuming, but given the characteristics of secondary HPT, IO-iPTH monitoring remains to be essential and important to confirm the sufficient removal of parathyroid glands. In conclusion, despite these factors, this criterion for the parathyroidectomy of secondary HPT is efficient and important.

In conclusion, iPTH value of <60 pg/mL (POD1) was a good predictor for successful parathyroidectomy. A 70% IO-iPTH drop from the baseline was appropriate to determine the sufficient removal of parathyroid glands during parathyroidectomy for patients with secondary HPT.
