# A Retrospective Study of the Impact of Intraoperative Intact Parathyroid Hormone Monitoring During Total Parathyroidectomy for Secondary Hyperparathyroidism: STARD Study: Erratum

**DOI:** 10.1097/01.md.0000471204.67061.d6

**Published:** 2015-08-14

In the article “A Retrospective Study of the Impact of Intraoperative Intact Parathyroid Hormone Monitoring During Total Parathyroidectomy for Secondary Hyperparathyroidism: STARD Study,^1^ which appeared in Volume 94, Issue 29 of *Medicine*, the word “data” appeared as a typo in one location in the abstract. The article has been corrected online.
